# Mr. Dooley on the Practice of Medicine

**Published:** 1901-08

**Authors:** 


### Mr. Dooley on the Practice of Medicine.

(From the *Buffalo Sunday News*, July 7th.)

"WHAT'S Christyan Science?" asked Mr. Hennessy.

"'Tis wan way iv gettin' th' money," said Mr. Dooley.

"But what's it like?" asked Mr. Hennessy.

"Well," said Mr. Dooley, "Ye have something th' matther with ye. Ye have a leg cut off."

"Th' Lord save us," exclaimed Mr. Hennessy.

"That is, ye think ye have," Mr. Dooley went on. "Ye think ye have a leg cut off. Ye see it goin' an' says ye to ye-ersilf: 'More expinse. A wooden leg.' Ye think ye've lost it. But ye're wrong. Ye're well as iver ye was. Both legs is attached to ye, on'y ye don't know it. Ye call up a christyan scientist or ye're wife does. Not manny men is christyan scientists, but near all women is, in wan way or another. Ye'er wife calls up a christyan scientist, an' says she: 'Me husband thinks he's lost a leg,' she says. 'Nonsense,' says th' christyan scientist, she says, f'r she's a woman, too. 'Nonsense,' says she. 'No man iver lost a leg,' she says. 'Well, 'tis sthrange,' says the wife. 'He's mislaid it thin,' she says, 'f'r he hasn't got it,' she says. 'He on'y thinks he's lost it,' says th' christyan scientist. 'Lave him think it on again,' she says. 'Lave him raymimber,' she says, 'they'se no such thing in th' wurruld,' she says, 'as pain an' injury,' she says. 'Lave him to put his mind hard to it,' she says, 'an' I'll put mine,' she says, an' we'll all put our minds to it, an' 'twill be all r-right,' she says. So she thinks an' th' wife thinks an' ye think th' best ye know how, an' afther awhile a leg comes peepin' out with a complete set iv tootsies, an' be th' time th' last' thought is expinded, ye have a set iv as well-matched gambs as ye iver wore to a picnic. But ye mustn't stop thinkin' or ye'er wife or th' christyan scientist. If wun iv ye laves go th' rope, th' leg 'll get discouraged an' quit growin.' Manny a man's sprouted a limb on'y to have it stop between th' ankle an' th' shin because th' christyan scientist was called away to see what ailed th' baby."

"Sure, 'tis all foolishness," said Mr. Hennessy.

"Well, sir, who can tell?" said Mr. Dooley. 'If it wasn't f'r medical progress, I'd be sure th' christyan scientists was wrong. But th' doctor who attinded me whin I was young 'd be thought as loonatical if he was alive today, as th' mos' christyan scientist that iver rayjooced a swellin' over a long distance tillyphone. He inthrajooced near th' whole parish into this life iv sin an' sorrow, he give us calomel with a shovel, bled us like a polis captain, an' niver thought anny medicine was good if it didn't choke ye goin' down. I can see him now as he come up dhriven' ol' gray an' yellow horse in a buggy. He had whiskers that he cud tie in a knot round his waist, an' him an' th' priest was th' on'y two men in th' neighborhood that carried a goold watch. He used to say 'twas th' healthiest parish in th' wurruld, barrin' hangins an' thransportations an' thim come in Father Hickey's province. Ivrybody thought he was a gr-eat man but they wudden't lave him threat a spavin in these days. He was catch-as-catch-can n' he'd tackle annything fr'm pneumony iv th' lungs to premachure baldness. He'd niver heerd iv mickrobes an' nather did I till a few years ago whin I was tol' they was a kind iv animals or bugs that crawled around in ye like spiders. I see pitchers iv thim in th' pa-apers with eyes like poached eggs till I dhreamed wan night I was a hayloft full iv bats. Thin th' dock down th' sthreet set me r-right. He says th' mickrobes is a vigitable an' ivry man is like a conservatory full iv millyons iv these potted plants. Some ar're good f'r ye an' some ar're bad. Whin th' chube roses an' geranyums is flourishin' an' liftin' their dainty petals to th' sun, ye're healthy, but whin th' other flower gets th' best iv these nosegays, 'tis time to call in a doctor. Th' doctor is a kind iv gardiner f'r ye. 'Tis his business f'r to encourage th' good mickrobes, makin' two pansies grow where wan grew befure an' to hoe out th' Canajeen thistle an' milk weed.

"Well, that sounds all r-right, an' I sind f'r a doctor. 'Dock,' says I, 'me vilets ar-re thinnin' out an' I feel as though I was full iv sage brush,' I say. Th' dock puts a glass chube in me mouth and says, 'Don't bite it.' 'D'ye think I'm a glass eater?' says I, talkin' through me teeth like a Kerry lawyer.' What's it f'r ?' I says. 'To take ye're timprachoor, says he. While I have th' chube in me mouth he jabs me thumb with a needle an' laves th' room. He comes back about th' time I'm r-ready to sthrangle an' removes th' chube. 'How high does she spout?' says I. 'Ninety-nine,' says he. 'Good hivens,' says I. 'Don't come near me, dock, or ye'll be sun sthruck,' I says. 'I've just examined ye'er blood,' he says. 'Ye're full iv weeds,' he says. Be that time I'm scared to death, an' I say a few prayers, whin he fixes a hose to me chest an' begins listenin'. 'Annything goin' on inside?' says I. ' 'Tis ye'er heart,' says he. 'Glory be,' says I. 'What's th' matther with that ol' ingine?' says I. 'I cud tell ye,' he says, but I'll have to call in Dock Vinthricle, th' specialist,' he says. 'I oughtn't be lookin' at ye'er heart at all, he says. I niver larned below th' chin an' I'd be fired be th' union if they knew I was wurrukin' on th' heart,' he says. So he sinds f'r Dock Vinthricle an' th' docks climbs me chest an' lishtens an' thin he says: 'They'se something th' matther with his lungs, too,' he says. 'At times they're full iv air, an' again,' he says, 'they ain't,' he says. 'Sind f'r Bellows,' he says. Bellows comes an' pounds me as though I was a roof he was shinglin', an' havin' accidentally hit me below th' belt, he sinds f'r Dock Laporattemy. Th' Dock sticks his finger into me as far as th' knuckle. What's that f'r?' says I. That's O'Hannigan's point,' he says. 'I don't see it,' says I. 'O'Hannigan must have had a fine sinse iv humor.' 'Did it hurt?' says he. 'Not,' says I, 'as much as though ye'd used an awl,' says I, 'or a chisel,' I says, 'but,' I says, 'it didn't tickle,' I says.

"He shakes his head an' goes out iv th' room with th' others an' they talk it over at tin dollars a minyit while I'm layin' there at two dollars a day—docked. Whin they come back, wan iv thim says: 'This here is a mos' inthrestin' case an' we must have th' whole class take a look into it,' he says. It means me, Hinnissy. 'Dock,' he says, 'ye will remove it's brain. Vinthricle, ye will have it's heart, an' Bellows, ye will take it's lungs. As f'r me,' he says, 'I will add wan more vermiform appendix to me belt,' he says. ' 'Tis sthrange how our foolish pre-decessors,' says he, 'niver got onto the dangers iv th' vermiform appendix,' he says. 'I have no doubt that that's what kilt Methausalem,' he says. So they mark out their wurruk on me with a piece iv red chalk an' if I get well, I look like a rag carpet. Sometime they lave things in ye, Hinnissy. I knowed a man wanst, Moriarty was his name—Tim Moriarty, an' he had to be hem-stitched hurridly because they was goin' to be a ball game that day an' they locked up in him two sponges, a saw, an ice pick, a goold watch an' a pair iv curlin' irons belongin' to wan iv th' nurses. He tol' me he didn't feel well, but he din't think anything iv it till he noticed that he jingled whin he walked.

That's what they do with ye nowadays, Hinnissy. Ivry time I go into Dock Cassidy's office, he gives me a look that makes me wisht I'd wore a suit iv chain armor. His eyes seem to say, 'Can I come in?' Between th' christyan scientists an' him 'tis a question iv whether ye want to be threated like a loonytic or like a can iv presarved vigitables. Father Kelly says th' styles iv medicine changes like th' styles iv hats. Whin he was a boy, they give ye quinine f'r whativer ailed ye, an' now they give ye sthrychnine an' nex' year they'll be givin' ye proosic acid, maybe. He says they're findin' new things th' matther with ye ivry day, an' ol' things that have to be taken out, ontil th' time is comin' whin not more thin half iv us'll be rale an' th' rest'll be rubber. He says they ought to enforce th' law iv assault with a deadly weepin' again th' doctors. He says that if they knew less about pizen an' more about gruel an' opened fewer patients an' more windows, they'd not be so manny christyan scientists. He says th' diff'rence between christyan scientists an' doctors is that christyan scientists thinks they'se no such thing as disease an' doctors thinks there ain't annything else. An' there ye ar-re."

"What d'ye think about it?" asked Mr. Hennessy.

"I think," said Mr. Dooley, "that if th' christyan scientists had some science an' th' doctors more christyanity, it wouldn't make anny diff'rence which ye called in—if ye had a good nurse."

